# The association between sporadic Legionnaires' disease and weather and environmental factors, Minnesota, 2011–2018

**DOI:** 10.1017/S0950268820001417

**Published:** 2020-06-29

**Authors:** J. K. Passer, R. N. Danila, E. S. Laine, K. J. Como-Sabetti, W. Tang, K. M. Searle

**Affiliations:** 1 University of Minnesota School of Public Health, Division of Epidemiology and Community Health, Minneapolis, Minnesota, USA; 2 Minnesota Department of Health, Infectious Disease Epidemiology, Prevention and Control, St. Paul, Minnesota, USA

**Keywords:** Epidemiology, geographical information systems, infectious disease control, Legionnaire's disease

## Abstract

From 2011 through 2018, there was a notable increase in sporadic Legionnaires' disease in the state of Minnesota. Sporadic cases are those not associated with a documented outbreak. Outbreak-related cases are typically associated with a common identified contaminated water system; sporadic cases typically do not have a common source that has been identified. Because of this, it is hypothesised that weather and environmental factors can be used as predictors of sporadic Legionnaires' disease. An ecological design was used with case report surveillance data from the state of Minnesota during 2011 through 2018. Over this 8-year period, there were 374 confirmed Legionnaires' disease cases included in the analysis. Precipitation, temperature and relative humidity (RH) data were collected from weather stations across the state. A Poisson regression analysis examined the risk of Legionnaires' disease associated with precipitation, temperature, RH, land-use and age. A lagged average 14-day precipitation had the strongest association with Legionnaires' disease (RR 2.5, CI 2.1–2.9), when accounting for temperature, RH, land-use and age. Temperature, RH and land-use also had statistically significant associations to Legionnaires' disease, but with smaller risk ratios. This study adds to the body of evidence that weather and environmental factors play an important role in the risk of sporadic Legionnaires' disease. This is an area that can be used to target additional research and prevention strategies.

## Background

**Fig. 1.**
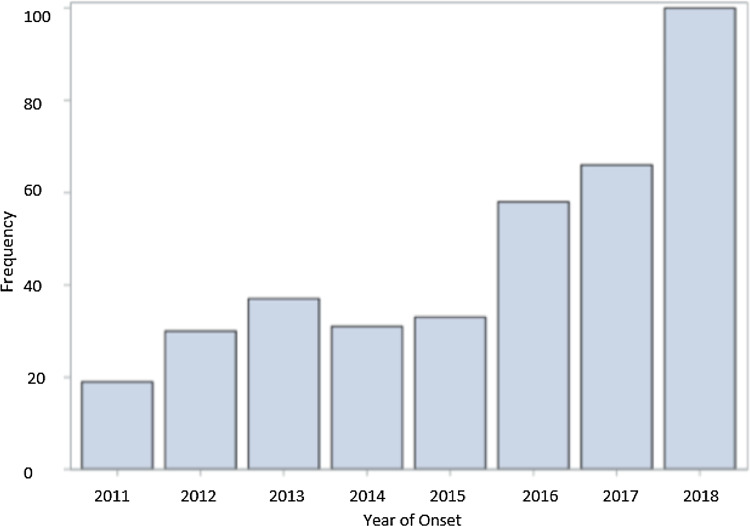
Yearly frequency (number of cases) of sporadic Legionnaires' disease cases from 2011 to 2018 in Minnesota.

*Legionella* is a Gram-negative bacterium found naturally in freshwater sources such as lakes, streams and reservoirs [1]. *Legionella pneumophila*, the primary disease-causing species of *Legionella*, is transmitted to humans when aerosolised droplets of contaminated water are inhaled or aspirated. As a result, legionellosis can occur, which primarily consists of Legionnaires' disease or Pontiac fever [1]. Extrapulmonary legionellosis can also occur, however is a much rarer form. Legionnaires' disease can lead to severe morbidity, hospitalization and death. The number of Legionnaires' disease cases in the USA has increased 5.5 times from 2000 to 2017 [2], and a similar increase was noted in Minnesota with cases increasing from 19 in 2011 to 100 in 2018 (Fig. 1).

*Legionella* bacteria live as an intracellular pathogen of freshwater protozoa and replicate within amoebae, making aquatic and soil ideal environmental reservoirs [1, 3]. The bacteria grow optimally in temperatures of 25–42 °C (77–108 °F) and these temperatures have been associated with increases in the risk of exposure and infection [4]. The incubation period for Legionnaires' disease is commonly identified as 2–10 days but ranges up to 26 days [5–7]. A 10–14 day incubation period is typically used when examining an outbreak to capture the range of potential exposures [8]. Outbreaks are usually linked to contaminated water systems [9]. These systems include showers, swimming pools, hot tubs, fountains, cooling towers, hot water heaters and large plumbing systems. Outbreaks are defined as two or more cases identified to have the same exposure source occurring within a 12-month period of each other, while sporadic cases include scattered cases that are not part of a known outbreak (e.g. do not come from the same known exposure source or do not have an identified exposure source). Weather and environmental factors can be used as predictors of sporadic Legionnaires' disease given the difficulty in identifying a common source for sporadic cases and can potentially be an area to target preventative measures [8, 10].

Legionnaires' disease is a required reportable disease in Minnesota. From surveillance conducted by the Minnesota Department of Health (MDH), there has been a recent increase in sporadic cases, notably during spring and summer months. The increase in sporadic cases suggests that factors related to weather and environmental changes may be playing an important role. Specifically, temperature, precipitation and humidity have previously been examined in various settings with mixed results [11–15]. To date, published studies examining the association between Legionnaires' disease and weather events have varying conclusions, primarily focused on outbreaks, and did not include ecological and geographic components. This study examined the associations between weather and Legionnaires' disease on a precise ecological and geographic scale using case reports and weather stations from across the state of Minnesota to capture the variability. It was hypothesised that weather and ecological factors are associated with sporadic Legionnaires' disease, specifically that increases in precipitation and temperature lead to an increased risk of sporadic Legionnaires' disease.

## Methods

### Case ascertainment

Case data were collected through required reporting from health care facilities, medical laboratories and all other licensed health care providers across the state of Minnesota between 2011 and 2018. Completeness of reporting is assured through annual audits of clinical laboratories conducting testing for Legionnaires' disease. Once case report forms were completed with medical record and demographic data, individual patients were contacted and interviewed regarding their exposures 10 days prior to the onset of symptoms per MDH protocols.

Data collected included patient name, residential address, type of residence, age, sex, race and ethnicity, date of symptom onset, discharge diagnosis, underlying health conditions, details of hospitalization including scans and the use of assisted respiratory devices, and laboratory-confirmed tests during visits of diagnosis. Data collected from interviews included locations and nights spent away from home; visits to hotels, casinos, conventions or public gatherings; exposure to water sources; use of respiratory equipment; exposure to construction projects; locations of work; and whether anyone else had similar illness around the same time. Residential addresses for each case were geocoded into latitude and longitude variables. These geocoded locations acted as a surrogate measure of location of exposure to *Legionella*.

To be a confirmed case, a patient had to have a positive *Legionella* laboratory test using a urine antigen, culture, paired serology or polymerase chain reaction (PCR) method [16]. Between 2011 and 2018, there were 651 confirmed cases. These included both pneumonic and non-pneumonic cases, with the vast majority being pneumonic. Since this analysis focused on sporadic cases and used household location as a surrogate for environmental exposure, outbreak- and travel-associated cases were identified and excluded. Travel-associated cases were defined as spending at least 1 night away from home within 10 days prior to symptom onset [17]. Outbreak cases were defined as two or more cases within the same 12-month time period with the same known exposure location, typically a spa/hot tub or other water feature identified during the outbreak investigation [18]. Cases with a post office (P.O.) box number, a missing home address or a missing date of symptom onset were removed. Four cases that spent the entire incubation period in a hospital were classified as definite nosocomial and excluded. This resulted in 374 eligible cases for this analysis. Of these cases, 348 cases were confirmed by urine antigen, 98 confirmed by culture and 58 confirmed by PCR (98 cases had multiple confirmatory tests). Serogroup for each case was unavailable for this analysis.

### Weather and ecological data

Weather and environmental data were collected using publicly available sources. Volunteers under the National Weather Service Cooperative Observers Program record daily weather observations for stations across Minnesota [19]. Daily temperature and precipitation data were collected from the National Weather Service reports provided by the Minnesota Department of Natural Resources [20]. Thirty stations across Minnesota had complete daily precipitation and temperature data from January 2011 through December 2018. The maximum daily temperature, daily precipitation and the latitude and longitude were acquired from all 30 stations during this 8-year time period. Addresses for the 374 cases were geocoded and spatially joined to their closest weather station based on their geographic location in ArcGIS v10.6.1 (ESRI, Redlands, CA, USA). This resulted in each case having unique temperature and precipitation variables for their residence location and time frame surrounding their symptom onset. Using the National Oceanic and Atmospheric Association, monthly average relative humidity (RH) data were collected at one centrally located station in St. Cloud, Minnesota [21].

To account for the full range of the incubation period, 14-day lag variables were created for average maximum temperature, total precipitation and average precipitation. These lagged variables were created by averaging or summing the daily weather variables for the 14 days prior to the first day of the onset of symptoms. Each case was assigned the monthly average RH based on the month of symptom onset. Each individual case had a variable for average maximum daily temperature for the 14 days prior to onset, total daily precipitation for the 14 days prior to onset, average daily precipitation for the 14 days prior to onset and average RH for the month of onset.

Using ArcGIS, land-use data from the US Geological Survey were matched to the latitude and longitude of each case [22]. Land-use across the state included cropland, deciduous needle-leaf forest, evergreen needle-leaf forest, grassland, mixed forest, permanent wetland, urban and built-up lands, and water bodies. Once a case was assigned a land-use variable, the variable was then dichotomised into either urban or rural (all non-urban areas) for analysis.

### Statistical analysis

Individual-level risk factors were explored and summary statistics were generated using SAS v9.4 (SAS Institute Inc., Cary, NC, USA). Data were checked for normality in distributions and two-sample *t* tests were used to identify if there were differences in the weather variables during the 14-day lag periods for days when there was a case and during 14-day lag periods for days when there was not a case. Data were aggregated by month and analysed longitudinally to examine changes in the risk of Legionnaires' disease over time and to determine the relationship between weather, environmental features and demographics at case locations using Poisson regression. For these analyses, cases were aggregated as counts per month. Weather variables were aggregated by calculating the mean of each value linked to the cases for that month, and the proportion of cases in an urban setting each month was calculated. The mean age and proportion of male cases were calculated for each month.

Unadjusted Poisson regression models examined the relationship between the incidence of sporadic Legionnaires' and 14-day total precipitation, 14-day average precipitation, 14-day average maximum temperature, monthly average RH and per cent of the cases that were in an urban setting. Adjusted Poisson regression models were developed to examine how the variables interact with each other and the outcome. Variables in the adjusted models included the weather and ecological variables that were in the previous unadjusted models along with mean age and proportion of males in each month. The final adjusted model contained average precipitation, average maximum temperature, monthly RH, proportion urban and mean age.

## Results

### Case summary statistics

There was an increase in Legionnaires' disease cases between 2011 and 2018 (Fig. 1). The majority of the case population was comprised of older, white males. The median age was 62 years, 67.2% were male and 86.5% were white (Table 1). The racial make-up of the study was similar to that of Minnesota (84.4% white in 2010). Four cases (1.1%) were 25 years old or younger, and 74 cases (19.8%) were 50 years old or younger. In addition, 59.4% of cases were either previous or current smokers, and 27.0% of cases had been diagnosed with chronic obstructive pulmonary disease, heart failure or both.

**Table 1.** Demographics for 374 reported cases between 2011 and 2018

	*N*	374	
Age (Years)***	Mean (s.d.)	62.0 (14.4)	
	Median (IQR)	62 (53–72)	
	Min	9	
	Max	97	
	Missing #	5	
Sex		*N*	%
	Male	250	67.2
	Female	122	32.8
	Missing	2	
Race		*N*	%
	White	321	86.5
	Black	32	8.6
	American Indian	6	1.6
	Asian	1	0.3
	Pacific	0	0.0
	Hispanic	1	0.3
	Multi-racial	5	1.4
	Unknown	5	1.4
	Missing #	3	0.8

*** P-Value: Alpha <.05.

### Weather summary statistics

**Fig. 2.**
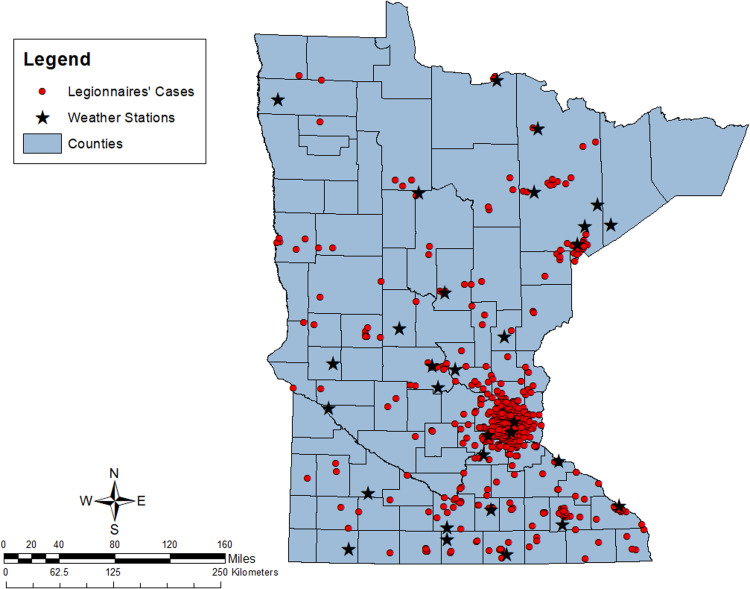
Map of Legionnaires' disease cases and data collecting weather stations for the state of Minnesota from 2011 to 2018.

Cases were spread across the state, with a concentration of cases in the Minneapolis-St. Paul metropolitan area (Fig. 2). The 30 weather stations cover the entire state, and each case had a weather station within a reasonable proximity. The mean distance from cases to a weather station was 12 miles (19 km), with a range of 0.2–98 miles (0.3–158 km) A Pearson's correlation coefficient showed a moderate correlation between average precipitation and average high temperature (*r*^2^ = 0.55). There was low correlation between average precipitation and RH (*r*^2^ = 0.16), and low correlation between average high temperature and RH (*r*^2^ = −0.03).

**Fig. 3.**
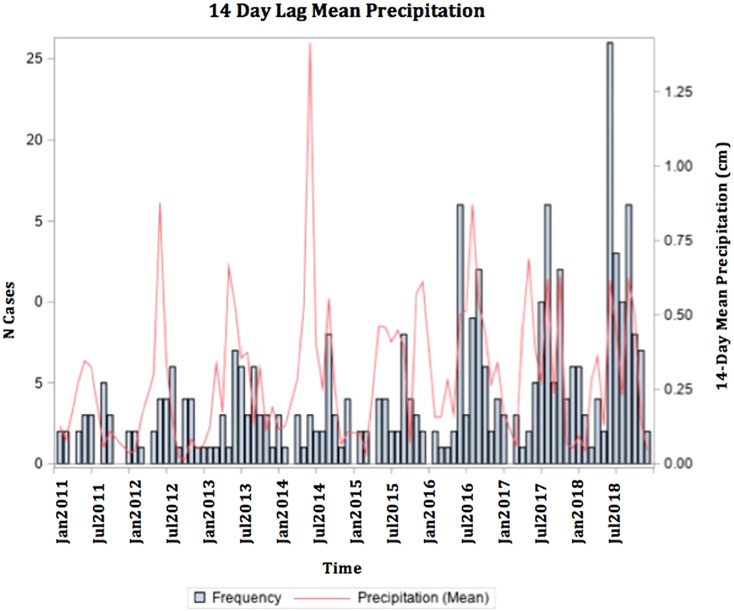
Frequency of Legionnaires' disease cases with 14-day mean precipitation of case onset date.

The average 14-day lagged total precipitation for all 374 cases was 4.96 cm (s.d. 3.13). The mean 14-day lagged average precipitation for cases was 0.39 cm (s.d. 0.24). The mean 14-day lagged average high temperature for all cases was 17.91 °C (s.d. 11.43) (64.24 (s.d. 20.13)°F). There was a seasonal trend in case occurrence, with 74.3% of cases falling between May and October (Fig. 3). During the peak season, the mean 14-day lagged average high temperature was 23.86 (s.d. 4.57)°C (74.95 (s.d. 7.32)°F).

### Statistical analysis

Days when cases occurred were associated with a 6.2 °C (11.1 °F) warmer 14-day lagged average high temperature and a 0.14 cm higher 14-day lagged average precipitation compared to days when a case did not occur. These associations were all statistically significant. There was no statistically significant difference in RH for the months that had cases compared to the months that did not have cases (Table 2).

**Table 2.** Two sample *t* test of the weather for the 14-day lag period for days that did have a case compared to days that did not have a case

	Case days (Mean (Range))	Non-case days (Mean (Range))	Difference (95% CI)	*P*-value
14-day average temperature (C)	17.91 (−16.39 to 32.22)	11.72 (−20.24 to 33.93)	6.19 (4.92–7.47)	<0.01
14-day average precipitation (cm)	0.39 (0.00–1.85)	0.25 (0.00–9.02)	0.14 (0.12–0.17)	<0.01
Monthly relative humidity (%)	72.76 (55.00–85.00)	69.82 (54.00–80.00)	2.95 (−1.22 to 7.11)	0.28

### Unadjusted Poisson regression results

In the unadjusted Poisson regression analyses, precipitation, temperature, RH and land-use all had statistically significant positive associations with the frequency of sporadic Legionnaires' disease (Table 3). When using 14-day lagged average precipitation as a continuous variable, for each 1 cm increase in 14-day average precipitation, there was a 4.32-fold increase in the risk of sporadic Legionnaires' disease (RR 4.32, CI 3.81–4.89; *P*-value <0.01) (Table 3). Using average precipitation as a categorical variable by quartiles, with each quartile increase, there was an increase in the risk of disease. With each 1 °C increase in 14-day lagged average high temperature, there was a 1.04-fold increase in the risk of disease (RR 1.04, CI 1.04–1.05; *P*-value <0.01) (Table 3). Each 1% increase in RH had a 1.04-fold increase in the risk of disease (RR 1.04, CI 1.04–1.05; *P*-value <0.01) (Table 3). Lastly, each 1% increase in urbanicity (per cent living in an urban setting) had a 1.37-fold increase in the risk of disease (RR 1.37, CI 1.18–1.59; *P*-value <0.01) (Table 3).

**Table 3.** Unadjusted Poisson regression models with 14-day lag

	RR*	95% CI**	*P*-value***
Precipitation total (cm)	1.12	1.11–1.13	<0.01
Precipitation average (cm)			
Continuous	4.32	3.81–4.90	<0.01
Categorical (Q2)	1.27	1.11–1.45	<0.01
Categorical (Q3)	2.26	2.00–2.55	<0.01
Categorical (Q4)	3.79	3.38–4.23	<0.01
Average high temperature (F)	1.03	1.02–1.03	<0.01
Monthly relative humidity (%)	1.04	1.04–1.05	<0.01
% Urban	1.37	1.18–1.59	<0.01

* RR: Risk Ratio.

** CI: Confidence Interval.

*** P-Value: Alpha <.05.

### Adjusted Poisson regression results

When accounting for other weather, ecologic and demographic factors, each 1 cm increase in 14-day average precipitation increased the risk of disease by 2.55-fold (RR 2.55, CI 2.18–2.99; *P*-value <0.01) (Table 4). In addition, with each 1 °C increase in average temperature, there was a 1.03-fold increase in the risk of disease (RR 1.03, CI 1.03–1.04; *P*-value <0.01) (Table 4). There was also a 1.04-fold increase in the risk of disease for every 1% increase in RH (RR 1.04, CI 1.03–1.05; *P*-value <0.01) (Table 4). Lastly, there was a 1.44-fold increase in the risk of disease for every 1% increase in urbanicity (RR 1.44, CI 1.21–1.72; *P*-value <0.01) (Table 4).

**Table 4.** Adjusted Poisson regression model with 14-day lag

Multivariate model	RR*	95% CI**	*P*-Value***
Average precipitation (cm)	2.46	2.10–2.89	<0.01
Average temperature (F)	1.02	1.02–1.02	<0.01
Monthly relative humidity (%)	1.04	1.03–1.05	<0.01
% Urban	1.44	1.09–1.71	<0.01
Mean age	1.00	0.99–1.01	0.49

* RR: Risk Ratio.

** CI: Confidence Interval.

*** P-Value: Alpha <.05.

## Discussion

Minnesota experienced an increase in sporadic Legionnaires' disease in the past 8 years with cases increasing from 19 in 2011 to 100 in 2018. Eighty per cent of the cases were above the age of 50 years, which aligns with the World Health Organization (WHO) published data that 75–80% of reported cases of Legionnaires' disease are over 50 years of age [23]. Additionally, a high percentage of cases had a history of cigarette smoking. This study population was largely white (86.5%); however, the distribution of race and ethnicity in this analysis was similar to that of Minnesota as a whole (84.4% white in 2010) [24]. There was a slightly higher proportion of male cases (67% male), which aligns with the WHO published data that 60–70% of reported cases are male [23]. The distribution of sex in Minnesota as a whole in 2010 was 49.6% male and 50.4% female [24].

We found a significant association between precipitation and sporadic Legionnaires' disease. Previous studies have used average precipitation rather than total precipitation for a lag period, and average 14-day precipitation was used as the more biologically plausible variable [25, 26]. Average maximum temperature, RH and urbanization, all had a statistically significant relationship with Legionnaires' disease occurrence as well. When average precipitation, maximum average temperature and RH were all analysed together, the magnitude of the risk ratio between average precipitation and Legionnaires' disease was reduced, signalling that temperature and RH may interact with precipitation. This could be due to the nature of RH being strongly associated with both precipitation and temperature, thus absorbing the direct associations observed in the univariate analyses when adjusted for other covariates. These findings are novel, and the interaction between RH, temperature and precipitation should be investigated further in their associations with sporadic Legionnaires' disease.

We also found that those who live in an urban setting are at an increased risk of sporadic Legionnaires' disease. Urbanization accounted for some of the location variability associated with both the weather and sporadic Legionnaires' disease. Urban settings are typically thought of as removed from ecologic exposures, but in many cases, are surrounded by lakes, rivers and environmental features where *Legionnella* can thrive. Lastly, age appeared to play a role in the risk of disease as the elderly population had the highest incidence of Legionnaires' disease. Overall, it is found that average precipitation had the strongest association to the risk of Legionnaires' disease.

These findings are consistent with previous work; however, most of those findings were based on outbreak-associated Legionnaires' rather than sporadic Legionnaires' disease. Hicks *et al*. found a strong association between Legionnaires' disease and precipitation; however, they did not account for RH or urbanization. Both Simmering *et al*. and Ricketts *et al*. found the strongest association to be temperature and RH [13, 27]. In our study, these two variables were also both found to have a statistically significant association with Legionnaires' disease. We measured precipitation and temperature at a precise scale, while Simmering *et al*. generalised the case date to a month of onset and used average weather variables for that month instead of using the exact onset date and weather variables for the 14-day incubation period. Ricketts *et al*. used daily weather variables similar to our analysis, but unlike our use of 30 weather stations, they used only one station to collect weather variables. Similarly, Karagiannis *et al*. used a single weather station for countrywide measures of temperature, humidity and precipitation [14].

We focused predominately on weather and environment using an ecologic study design, but additional methods to analyse the relationship between weather and Legionnaires' disease have been used. Fisman *et al*. found an association between increased incidence of disease with precipitation and humidity, but not temperature using a case-crossover approach [28]. A study examining 29 years of Legionnaires' disease found that after adjusting for a seasonal effect, there was an increase in odds of Legionnaires' disease with a decrease in watershed levels and a decrease of lake temperatures 4 weeks prior to the onset of symptoms, rather than any weather effects, in Toronto, Ontario [29]. Other studies looked at windspeed, or used dewpoint or atmospheric pressure instead of humidity, which may contribute to slightly varied results [12, 28, 30].

When accounting for weather and environmental factors, this analysis was at a larger scale with higher resolution than previous studies. We used data from 30 weather stations geographically distributed across the state of Minnesota to account for local variation in weather, which increased precision and accuracy. Geocoding household locations to their closest weather station gave each case a unique and more precise temperature and precipitation measurement during their exposure period. The household location acted as a surrogate for the location of exposure, as people spend most of their time at or near this location. The 8 years of data were an additional strength of our study because it allowed for the assessment of trends over time for a meaningful number of cases. In addition, demographics and land-use were assessed as possible risk factors for sporadic Legionnaires' disease, setting this analysis apart from others.

One of the limitations of our study was using an ecological study design. The data included individuals who were diagnosed with Legionnaires' disease, but not a comparison population. Another limitation was that due to a lack of publicly downloadable data, monthly RH were collected at one central location and generalised to all cases, making it less precise. In addition, the precipitation variable used in this analysis included both rain and snow. Time periods with snow instead of rain could have partially explained why when accounting for temperature, precipitation had less of an association. Some areas of the state may have had better access to care or have healthier living environments. We were not able to account for these factors, nor were we able to account for the travelling that individuals may have done during the day or possible exposures away from their home; however, it was assumed that any daily travel was close enough to the residential address to have similar weather conditions. Finally, those with a P.O. box were also excluded, which may have excluded some rural populations.

## Conclusion

We found that weather and ecology were strongly associated with sporadic Legionnaires' disease in Minnesota from 2011 to 2018. These findings show that there is an increased risk of sporadic Legionnaires' disease with increased precipitation. Higher precipitation can lead to more saturated soil, which increases the risk of contamination with *Legionella* [4]. During wetter time periods, prevention methods for water and soil contamination could be targeted to reduce potential exposures. Health care professionals should also be aware of these time periods when individuals are at higher risk so individuals, particularly those at highest risk, can be diagnosed quickly and treated timely to prevent the development of serious complications due to sporadic Legionnaires' disease.

## Data Availability

The data that support the findings of this study are available by request to the corresponding author.
